# F205. OLFACTORY IDENTIFICATION IN 7-YEAR OLD CHILDREN AT FAMILIAL RISK TO DEVELOP SCHIZOPHRENIA

**DOI:** 10.1093/schbul/sby017.736

**Published:** 2018-04-01

**Authors:** Anna Hester Ver Loren van Themaat, Jens Richardt Møllegaard Jepsen, Camilla Christiani, Merete Nordentoft

**Affiliations:** 1 University of Copenhagen; 2 Mental Health Services – Capital Region of Denmark, Child and Adolescent Mental Health Centre, The Lundbeck Foundation Initiative for Integrative Psychiatric Research, Center for Clinical Intervention and Neuropsychiatric Schizophrenia Research; 3 Mental Health Centre Copenhagen

## Abstract

**Background:**

Olfactory dysfunction has repeatedly been observed in individuals diagnosed with schizophrenia. The most stable and consistent finding on the behavioral level is that of smell identification deficits. However, the nature of olfactory identification abnormalities seems to extend to structural abnormalities in the underlying neurobiology of the olfactory system. Furthermore, smell identification deficits are also documented in first-episode patients and non-psychotic first-degree relatives of schizophrenia patients. Family members of schizophrenia patients also show structural abnormalities of the olfactory system, suggesting that these may serve as an endophenotype for the development of schizophrenia.

Only a few studies examined the olfactory identification ability in adolescents at-risk for schizophrenia and suggested smell identification deficits as a risk marker for schizophrenia. These studies included adolescents at clinical as well as at genetic risk for schizophrenia. None of these studies focused on children at genetic risk for schizophrenia. Therefore, we investigated the olfactory identification ability in children of parents with schizophrenia in comparison to children of parents without a psychotic disorder. As we are also interested in the specificity of the olfactory impairments to schizophrenia, we included children of parents with bipolar disorder. We hypothesize that children at genetic risk for schizophrenia would have the most severe smell identification deficits and that children of bipolar disorder patients would have less severe deficits than the at-risk for schizophrenia group but more severe than the group of children without a psychotic parent.

**Methods:**

Participants - The olfactory identification ability was assessed in 202 children of schizophrenia patients (‘children at familial risk for schizophrenia’) in relation to that of 200 children of parents without a psychotic disorder (‘controls’). In addition, we also assessed the B-SIT in 120 children of bipolar disorder patients (‘children at familial risk for bipolar disorder’). All children were 7 years of age at the time of assessment and they were part of the Danish High Risk and Resilience Study – VIA7.

Brief Smell Identification Test - The Brief Smell Identification Test (B-SIT) contains 12 items that need to be scratched and sniffed. The test has excellent reliability (> 0.80) and demonstrates agreement for abnormal olfaction comparing B-SIT with the San Diego Odor Identification Test (SDOIT). A maximum score of 12 reflects intact olfactory identification functioning. B-SIT has been conducted in patients with neurodegenerative disorders (Parkinson’s disease and Alzheimer’s disease) and can be used for individuals above 5 years of age.

Statistics - We will use analysis of covariance (ANCOVA) for analysis of the B-SIT total scores with ‘diagnosis of parent’ as the independent variable and age and sex as covariates for the three groups.

**Results:**

Analyses will be performed within the next 3 months so can be presented in April 2018.

**Discussion:**

Conclusion and discussion cannot be drawn at this time.

